# Random Phenotypic Variation of Yeast (*Saccharomyces cerevisiae*) Single-Gene Knockouts Fits a Double Pareto-Lognormal Distribution

**DOI:** 10.1371/journal.pone.0048964

**Published:** 2012-11-06

**Authors:** John H. Graham, Daniel T. Robb, Amy R. Poe

**Affiliations:** 1 Department of Biology, Berry College, Mount Berry, Georgia, United States of America; 2 Department of Physics, Astronomy, and Geology, Berry College, Mount Berry, Georgia, United States of America; 3 Department of Mathematics, Computer Science and Physics, Roanoke College, Salem, Virginia, United States of America; 4 Center for Integrative Genomics, Georgia Institute of Technology, Atlanta, Georgia, United States of America; CRS4, Italy

## Abstract

**Background:**

Distributed robustness is thought to influence the buffering of random phenotypic variation through the scale-free topology of gene regulatory, metabolic, and protein-protein interaction networks. If this hypothesis is true, then the phenotypic response to the perturbation of particular nodes in such a network should be proportional to the number of links those nodes make with neighboring nodes. This suggests a probability distribution approximating an inverse power-law of random phenotypic variation. Zero phenotypic variation, however, is impossible, because random molecular and cellular processes are essential to normal development. Consequently, a more realistic distribution should have a y-intercept close to zero in the lower tail, a mode greater than zero, and a long (fat) upper tail. The double Pareto-lognormal (DPLN) distribution is an ideal candidate distribution. It consists of a mixture of a lognormal body and upper and lower power-law tails.

**Objective and Methods:**

If our assumptions are true, the DPLN distribution should provide a better fit to random phenotypic variation in a large series of single-gene knockout lines than other skewed or symmetrical distributions. We fit a large published data set of single-gene knockout lines in *Saccharomyces cerevisiae* to seven different probability distributions: DPLN, right Pareto-lognormal (RPLN), left Pareto-lognormal (LPLN), normal, lognormal, exponential, and Pareto. The best model was judged by the Akaike Information Criterion (*AIC*).

**Results:**

Phenotypic variation among gene knockouts in *S. cerevisiae* fits a double Pareto-lognormal (DPLN) distribution better than any of the alternative distributions, including the right Pareto-lognormal and lognormal distributions.

**Conclusions and Significance:**

A DPLN distribution is consistent with the hypothesis that developmental stability is mediated, in part, by distributed robustness, the resilience of gene regulatory, metabolic, and protein-protein interaction networks. Alternatively, multiplicative cell growth, and the mixing of lognormal distributions having different variances, may generate a DPLN distribution.

## Introduction

Developmental homeostasis and robustness are related concepts having very different histories. Developmental homeostasis, the older of the two concepts, has two independent aspects: canalization and developmental stability [1], [2]. Canalization is the stability of development under different environmental and genetic conditions, while developmental stability is the stability of development under constant environmental and genetic conditions [3]. Robustness, a more recent concept rooted in systems biology, is reduced sensitivity to genetic and environmental perturbations [4], [5], [6], [7]. Such perturbations include 1) genetic changes, 2) systematic changes in the external environment, and 3) stochastic fluctuations of the internal or external environment [5]. Developmental stability is thus a subcategory of robustness. Despite considerable interest in both developmental stability and robustness, their genetic architectures are largely unknown [5], [6], [8], [9], [10], [11].

Developmental stability is thought to be mediated by heterozygosity [12], [13], genomic coadaptation [12], [14], and stress proteins such as *Hsp90* [15], [16], [17], [18]. Robustness, on the other hand, is thought to be influenced by the topology of gene-interaction networks (distributed robustness) and genetic redundancy [5], [19]. These differences reflect different research histories more than any real differences in causation: two different ways of looking at the problem. In this paper, we focus on the predicted effects of distributed robustness on the statistical distribution of developmental instability and random phenotypic variation (lack of robustness).

Distributed robustness involves the complexity of gene regulatory, metabolic, and protein-protein interaction networks. If a link in a network is broken, it may (in many cases) be bypassed with little impact on fitness [20], [21]. Wagner and colleagues [19], [22] believe distributed robustness is more important than redundancy, which involves duplicate genes. If there are two or more identical copies of a particular gene, inactivating one of them will have a minimal impact on fitness.

If distributed robustness is the main contributor to developmental stability, then the topology of interactions among genes, proteins, and metabolites should be critically important (but see [23]). The degree distributions of such interaction networks are said to approximate an inverse power-law distribution [24] (but see [25], [26], [27], [28]), *P*(*k*)≈*k* ^−γ^, where *P*(*k*) is the probability that a node (or vertex) has *k* links (or edges) and γ is a coefficient that reflects the declining frequency as *k* increases. Inverse power-law distributions are monotonically decreasing and they have long (fat) tails. Other proposed distributions, such as truncated power-law and stretched exponential distributions, also suggest scale-free behavior, but only over a part of the network [25]. These are also consistent with distributed robustness.

Assuming that distributed robustness contributes to developmental stability and robustness, what should the distribution of random phenotypic variation (i.e., developmental instability) look like? Perturbing a highly connected node (i.e., a hub) has a greater phenotypic effect than perturbing a node with only a few links [29]. The simplest possible assumption is that the response *R* to perturbation of a particular node (or link) in a gene regulatory, metabolic, or protein-protein interaction network is proportional to the node's connectedness, *k*. This is true for protein-protein interactions involving single-copy genes and synthetic lethal interactions involving all genes [29]. Assuming a simple inverse power-law distribution, the probability distribution of the response *R* to a random perturbation will then be given by *P*(*R*)≈*R* ^−γ^. In addition, assume that random developmental variation is a normal (and necessary) component of development, such that zero variation is impossible for continuous traits [30]. If both assumptions hold, then the expected distribution of developmental instability resulting from single-gene knockouts should have these characteristics: (1) no populations should be composed entirely of perfectly symmetrical (or uniform) individuals, and (2) the distribution should have a fat upper tail due to the effect of network topology. A distribution fitting these criteria is the double Pareto-lognormal distribution (DPLN), a mixture distribution introduced into the study of developmental instability by Babbitt et al. [31]. Various complex networks and natural phenomena exhibit a DPLN distribution [32]. The abundances of mRNA, proteins, and metabolites, for example, fit a DPLN distribution [33].

The Pareto distribution is the name given to a cumulative distribution function that has a power-law tail. In the context of networks, the value of the Pareto cumulative distribution function is the number of nodes having degree greater than *k* [34]. A power-law probability distribution function, in contrast, gives the number of nodes whose degree is exactly *k*. The power-law then is the probability density function associated with the cumulative distribution function given by Pareto's law. Both have fat upper tails. Gene expression data sets in yeast, mouse, and human cells follow a Pareto-like probability distribution [35].

Alternative distributions include the right and left Pareto-lognormal distributions, as well as the normal, lognormal, Pareto, and exponential distributions. The right-handed Pareto-lognormal (RPLN) distribution resembles the DPLN, but has a fat upper tail and a lognormal lower tail [36]. Given our two assumptions, it should provide as good, or better, a fit as the DPLN, since we have no *a priori* reason to expect a fat lower tail. The left Pareto-lognormal (LPLN) distribution, on the other hand, lacks a fat upper tail (it has a fat lower tail) [36] and we do not expect this to fit well. If the response to major perturbation of a node is proportional to the node's connectedness, but there is little or no additional developmental noise (minor perturbations), then we would expect the Pareto distribution to provide the best fit. If neither assumption is true, then we might expect a normal distribution (if errors are additive), a lognormal distribution (if errors are multiplicative), or an exponential distribution (if perturbations fit an exponential distribution).

*Saccharomyces cerevisiae* (Baker's yeast) is an ideal species in which to examine the predictions of network topology and developmental instability. Its genome has been sequenced and the degree distributions of its metabolic, protein-protein interaction, and gene regulatory networks roughly approximate the predicted inverse power-law distribution [10], [37], [38], [39] (but see [25]). Moreover, phenotypic variation of single-copy gene knockouts increases with both protein-protein interaction degree and synthetic-lethal interaction degree (see Figure 3B and 3D in [29]). And finally, published data are readily available. Here, we show that random phenotypic variation of haploid single-gene knockouts in *S. cerevisiae* fits a double Pareto-lognormal distribution better than several other skewed and symmetrical distributions.

## Materials and Methods

### Yeast Data Set

Working with 4,718 strains of haploid single-gene knockouts [40], Levy and Siegal [29] estimated the overall phenotypic variance resulting from single deleted genes, which represent a kind of major genetic perturbation [5]. They called this the phenotypic potential, which is equivalent to the variation among clone mates in a common environment, an alternate estimator of developmental instability. We used Levy and Siegal's estimates of phenotypic potential (PP) from Table S1 in [29]. Yeast phenotypes are described by Ohya et al. [40]. They include long-axis length of the mother nucleus, long-axis length of the cell, maximal distance between actin patches, and bud angle.

### Statistical Models

The DPLN is a mixture distribution [36]. The left and right tails are Pareto distributions, which have fat tails, whereas the body of the distribution is lognormal. The parameters of the DPLN distribution are the lognormal mean (*ν*) and variance (*τ* ^2^), and power-law scaling exponents for the right (*α*) and left tails (*β*). The probability density function, *dPlN* (*α*, *β*, *ν*, *τ* ^2^), is
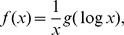
where *g*(*y*) is a normal-Laplace distribution
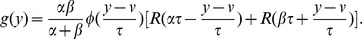

*R*(*z*) is the Mill's ratio, 
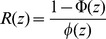
, where Φ is the cumulative density function and *φ* is the probability density function for the standard normal distribution *N*(0,1). (See Appendix S1 for corrections to four of the equations in Reed [36].)

We fitted the phenotypic potential of *S. cerevisiae* single-gene knockouts to DPLN, right Pareto lognormal (RPLN), left Pareto lognormal (LPLN), normal, lognormal, Pareto, and exponential distributions. For the normal, lognormal, Pareto, and exponential distributions, we used maximum likelihood estimators of the parameters (e.g., mean and variance for the normal distribution). For the DPLN, RPLN, and LPLN distributions, we used two independent algorithms, the Downhill Simplex Method in Multidimensions (section 10.4 in [41]) and Direction Set (Powell's) Methods in Multidimensions (section 10.5 in [41]), to carry out the maximization of the log-likelihood function. Both algorithms gave essentially identical parameters for all three distributions.

### Model Selection

We used the Akaike Information Criterion (*AIC*) [42], [43] to select the best model from among DPLN, RPLN, LPLN, normal, lognormal, Pareto, and exponential distributions. *AIC* is a measure of the relative goodness of fit of a statistical model. The models are ranked by their *AIC* values, where *AIC* = −2 ln(*L*)+2*d*. ln(*L*) is the value of the log likelihood function for a particular model, while *d* is the number of parameters in a model. A corrected version for finite sample sizes is 
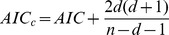
, where *n* = the sample size. The smaller the *AIC* value for a distribution, the more likely it is that the distribution fits the data the best. Because *AIC* values are relative, the *AIC* differences (Δ*_i_*) are calculated: 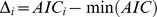, where min(*AIC*) is the smallest *AIC* value among all of the models. *AIC* is estimated for each of *i* models. Akaike weights (*w_i_*) reflect the normalized likelihood of the models given the data [44], 
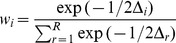
.

## Results

**Figure 1:**
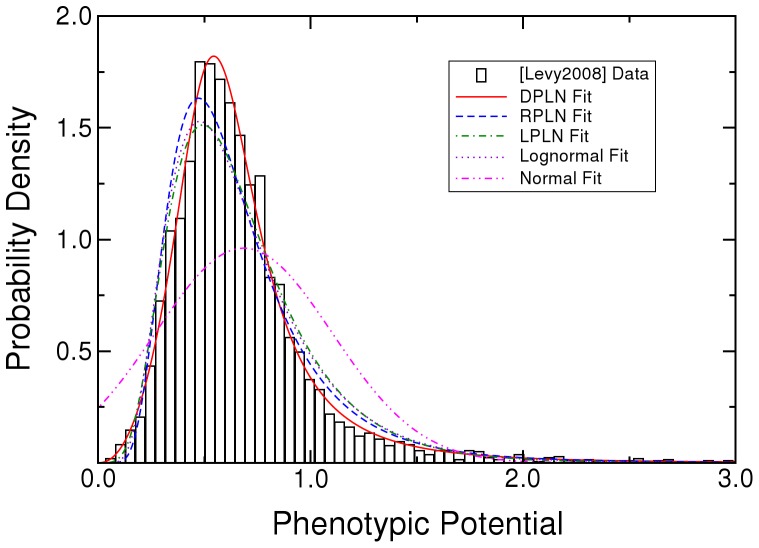
Probability distributions fit to a histogram of random phenotypic variation (phenotypic potential) in *Saccharomyces cerevisiae* gene knockouts. Histogram data are from Table S1 in [29]. DPLN is the double Pareto-lognormal distribution. RPLN is the right Pareto-lognormal distribution. LPLN is the left Pareto-lognormal distribution. Simple Pareto and exponential distributions omitted.

**Figure 2:**
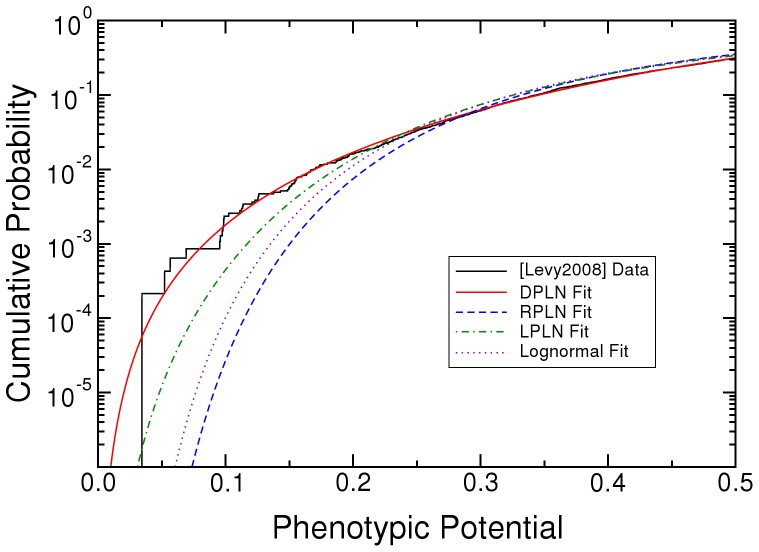
Lower tail of the cumulative distribution function (cdf) of random phenotypic variation (phenotypic potential) in *Saccharomyces cerevisiae* gene knockouts, and the DPLN, RPLN, LPLN, and lognormal fits. Data are from Table S1 in [29]. DPLN is the double Pareto-lognormal distribution. RPLN is the right Pareto-lognormal distribution. LPLN is the left Pareto-lognormal distribution. Simple Pareto and exponential distributions omitted.

The phenotypic potential of *S. cerevisiae* fits a DPLN distribution better than RPLN, LPLN, normal, lognormal, exponential, or Pareto distributions (Figure 1, and Tables 1 and 2). The superior fit of the DPLN is especially noticeable in the cumulative distribution function of the lower tail (Figure 2). The relative probability of the RPLN, the next best distribution, is 5.5×10^−65^. Having almost 5000 data points means that we can be extremely confident of the DPLN for the yeast data, even though three other distributions (RPLN, LPLN, lognormal) look close by eye.

**Table 1:** The *AIC*_c_ values for the fit of seven distributions to the phenotypic potential data from *Saccharomyces cerevisiae*.

Distribution	log(*L*)	*d*	*AIC*_c_	Δ_i_	*w*_i_
DPLN	−713.77	4	1435.55	0.0	1
RPLN	−862.73	3	1731.47	295.92	5.5×10^−65^
LPLN	−888.88	3	1783.76	348.21	2.4×10^−76^
Lognormal	−902.96	2	1809.93	374.39	5.0×10^−82^
Normal	−2525.41	2	5054.82	3619.27	0
Exponential	−2912.06	1	5826.13	4390.58	0
Pareto	−7478.32	2	14960.64	13525.09	0

Log(*L*) is the log-likelihood function. *d* is the number of parameters. *AIC*_c_ is the corrected Akaike Information Criterion (*AIC*). The rescaled *AIC*c is Δ_i_ and the Akaike weights are *w*_i_. DPLN is the double Pareto-lognormal distribution. RPLN is the right Pareto-lognormal distribution. LPLN is the left Pareto-lognormal distribution. The sample size *n* was 4,680. Data is from Table S1 in [29].

**Table 2:** Parameter estimates for the fit of seven distributions to the phenotypic potential data from *Saccharomyces cerevisiae*.

Distribution	Parameters
DPLN	α = 3.141, β = 3.242, τ = 0.1909, ν = −0.5121
RPLN	α = 4.124, τ = 0.4165, ν = −0.7446
LPLN	β = 5.198, τ = 0.4432, ν = −0.3098
Lognormal	τ = 0.4849, ν = −0.5021
Normal	σ = 0.4151, μ = 0.6854
Exponential	α = 1.459
Pareto	α = 0.3328, x_m_ = 0.03

DPLN is the double Pareto-lognormal distribution. RPLN is the right Pareto-lognormal distribution. LPLN is the left Pareto-lognormal distribution. Data is from Table S1 in [29].

There are two major differences between the DPLN and the RPLN, LPLN, and lognormal distributions (Figure 1). The DPLN has fewer nodes (i.e., single-gene knockouts) having low phenotypic potential; the mode of the DPLN is shifted to the right of that of the RPLN, LPLN, and lognormal. The DPLN, however, has more nodes, simultaneously, in both tails of the distribution (see Figure 2 for the lower tail).

## Discussion

The robustness of living organisms is thought to arise from redundancy and distributed robustness [4], [22]. Redundancy involves duplicate copies of genes. Distributed robustness involves the topology of gene regulatory, metabolic, and protein-protein interaction networks. These networks typically resemble scale-free networks [24], [45], [46], at least in part [25], and they are robust to perturbation [47], [48]. But a metabolic pathway's fragility lies in the highly connected nodes, or hubs, in this network. Error tolerance comes at a price [49]. Knock out a highly connected node and the system fails.

Networks consist of nodes and links [50]. In a metabolic network, the nodes are chemical intermediates (substrates and products), and the links are enzymatically mediated reactions (enzymes). In a protein-protein interaction network, the nodes are individual proteins and the links are their binding relationships with other proteins. In a gene regulatory network (protein-DNA interactome), which is a directed network, the nodes are genes, which interact through transcription factors, chromatin regulatory proteins, and other DNA-binding molecules. In such a network, one can distinguish in-degree and out-degree distributions. The in-degree of a gene (or node) represents the number of other genes influencing that particular gene, while its out-degree represents the number of other genes that it influences.

A highly connected node is a hub. In many biological networks, or parts of these networks, the connectivity *P*(*k*) of nodes follows a power law, *P*(*k*)≈*k* ^−γ^, distribution. Most nodes have few links, but a few hubs may have hundreds or thousands of links. The hubs connect the less connected nodes to the system. These systems are typically scale-free [45], [51] and hierarchical [52]. Most perturbations should have little effect on organism-wide developmental instability, unless they perturb a hub [9], [29].

Other degree distributions have also been fitted to biological networks, including truncated power-law, exponential, and stretched exponential distributions [25]. The truncated power-law distribution resembles a power-law distribution, followed by a sharp drop off. It fits yeast co-expression networks [25]. A stretched exponential has a power-law exponent inserted into an exponential function. It fits protein-protein interaction networks of *Drosophila* and *Caenorhabditis* [53]. Purely exponential distributions fit some network data too. For example, the in-degree of some gene-regulatory networks follows an exponential distribution, while the out-degree follows a scale-free distribution [54], [55], [56]. Nevertheless, these other distributions are qualitatively similar to inverse power-law distributions (few hubs and many nodes having few links) [25]. Consequently, these degree distributions are still consistent with distributed robustness.

In yeast, *Saccharomyces cerevisiae*, the balanced distribution of nodes and hubs buffers phenotypic variation [29]. (Buffering refers to the ability of a system to minimize, or soften, perturbations [9].) Most single-gene knockouts have almost no effect on phenotypic variation because they are not hubs. According to Levy and Siegal [29], approximately 300 gene products are responsible for most of the phenotypic variation when they are knocked out. These are phenotypic capacitors, genetic elements whose mutation serves as a major perturbation, reducing genetic robustness and increasing heritable phenotypic variation [5]. When the source of the increased variation is non-genetic, Masel and Siegal [5] refer to these as phenotypic stabilizers. In yeast, these 300 capacitors (or stabilizers) are predominantly single-copy hubs.

Our results, the close fit to the DPLN, are consistent with the hypothesis that network topology, and hence distributed robustness, plays a role in developmental stability. Nevertheless, it is unlikely to play the only role. Levy and Siegal [29] also found that many hubs in *S. cerevisiae* genetic networks exist in multiple copies, which would blunt the effect of a mutation in just one copy. Consequently, capacitors of phenotypic variation are more likely to be single-copy hubs. But even these single-copy hubs are likely to be idiosyncratic capacitors. Based upon evolutionary simulations of gene-regulatory networks, Siegal et al. [23] argue that network topology is only a weak predictor of the response to perturbation. Given the uncertain, and complicated, role of network topology, other, unknown, influences may be responsible for, or contribute to, the power-law behavior in the upper and lower tails of the distribution. For example, the DPLN emerges in the size distributions of cities. According to Reed [57] and Giesen et al. [58], the DPLN distribution is the steady-state of a stochastic urban growth process, with random city formation. It can also arise from a continuous mixture of lognormal distributions having different variances [31]. Similar processes can be easily envisioned in cell growth, which is inherently a multiplicative process. Multiplicative errors, which generate lognormal distributions, occur whenever growth is active, which is whenever cytoplasm at time *t*−1 actively participates in the production of cytoplasm at time *t* [59].

Lu and King [33] have speculated that the DPLN distribution of abundances of mRNAs, proteins, and metabolites may be a consequence of multiplicative error, which is ubiquitous in biological systems. They argue that independent multiplicative processes contribute to the central lognormal part of the distribution, while mutually dependent multiplicative processes contribute to the power-law tails. They posit that positive feedback and network topology are the most likely interactions generating the tails.

In addition to these alternative explanations for the DPLN, we have not accounted for the better fit to the DPLN over the RPLN. The lower tail of the distribution of phenotypic potential appears to fit a power-law distribution, but with a positive slope. Allometric relations, such as the scaling of metabolic rate with mass, are the best-known scaling relationships having a positive slope [60], [61]. At the lower end of the DPLN distribution, below a phenotypic potential of 0.6, random molecular and sub-cellular noise maintains a background level of variation, which network buffering effectively keeps under control. This might occur if the weak links within gene regulatory, metabolic, and protein-protein interaction networks are doing most of the buffering [62]. Alternatively, we are simply looking at a mixture of lognormal distributions having different variances.

Other researchers have examined the statistical distribution of developmental errors, but have done so with radially or bilaterally symmetrical traits in natural populations of multicellular organisms. Van Dongen and Møller [63], for example, examined random developmental variation (fluctuating asymmetry) in flower petals, ray flowers, and bird tails. They studied multiple petals and ray flowers from individual plants, and tail feathers from consecutive molts of individual birds. They found that the normal distribution was a good approximation to the distribution of developmental noise among random genotypes within these three, presumably outbred, populations. The yeast knockouts in the Levy-Siegal study [29], however, are not a random sample of genotypes from a natural population; they are a random sample of single-gene knockouts having a homogeneous genetic background. It will be informative to have both kinds of studies, since they represent the extreme ends of a continuum.

How does the DPLN alter our understanding of networks and organismal evolution? All yeast single-gene knockouts (or loss-of-function mutations) are heritable, by definition, but not all of the phenotypic variation generated by such knockouts is heritable. Most knockouts barely increase phenotypic variation beyond the cloud of random, non-heritable, developmental noise. This is the variation generating the lower tail of the DPLN. The knockouts in the upper tail of the distribution, however, represent heritable variation in developmental noise. Such heritable variation should be accessible to natural selection, which could then fine-tune developmental noise to maximize fitness. Consequently, by understanding the complex relationships between gene regulatory, metabolic, and protein-protein interaction networks and phenotypic variation, we may eventually begin to understand why organisms are not less variable (or more variable) than they already are.

The close fit of phenotypic variation in single-gene knockouts of yeast to the DPLN distribution suggests that disruption of most nodes has a minor, but significant, impact on phenotypic variation. This impact is greater than one would expect from the RPLN, LPLN, and lognormal distributions. Consequently, the DPLN distribution suggests that more random phenotypic variation is potentially heritable than one would expect under, say, a lognormal distribution, or less random phenotypic variation is heritable than one would expect under a normal distribution.

The generality of our results will have to await further research on random phenotypic variation of gene deletion and RNAi lines of multicellular organisms, such as *Arabidopsis thaliana*, *Drosophila melanogaster*, and multicellular colonies of *S. cerevisiae*. The gene deletion and RNAi lines exist, and the methods of estimating random phenotypic variation in plants [64], [65], [66] and animals [9] are well developed, using the methods of fluctuating asymmetry [9]. In addition, Raz et al. [67] recently showed how to apply methods of fluctuating asymmetry to colonies of microorganisms. Unfortunately, however, the phenotypic data sets for these lines do not exist at this time.

An obvious extension of our study to multicellular organisms should begin with *S. cerevisiae*. Yeast are unicellular eukaryotes, but colonies on agar plates behave somewhat like multicellular organisms. Palkova and colleagues [68] have studied the relationship between variation at the unicellular and multicellular (colonial) levels in yeast. Knocking out the *CCR4* gene increases phenotypic variation among cells and also increases the irregularity of entire colonies [69]. This suggests a possible linkage between cellular and multicellular variation, at least for this gene in this species.

In conclusion, we have demonstrated that the DPLN fits the distribution of random phenotypic variation of yeast single-gene knockouts better than several competing distributions. This result is consistent with the hypothesis that distributed robustness operating in a noisy developmental system buffers phenotypic variation, at least in part. It is also consistent with the hypothesis that the DPLN arises from multiplicative cell (or cytoplasmic) growth and the mixing of lognormal distributions having different variances. Moreover, these hypotheses, one a biological hypothesis and the other a statistical hypothesis, are not mutually exclusive. Further research will be necessary to distinguish between them. Finally, it will be important to refine the behavior of the DPLN for future models of phenotypic variation. For example, how will the DPLN change if the nodes experience only minor perturbation? Will it approach a lognormal distribution instead, as *α* and *β* approach infinity? And what will the distribution of phenotypic variation look like in a population of yeast in which each clone is a product of sexual reproduction? Will it approach the lognormal distribution? Or will it fit the normal distribution, as Van Dongen and Moller [63] suggest for flowering plants and birds?

## Supporting Information

Appendix S1 Errata in the original article on the double Pareto-lognormal distribution by Reed.
(DOCX)

## References

[1] Waddington CH (1942) Canalization of development and the inheritance of acquired characters. Nature 150: 563–565. 10.1038/1831654a0 13666847

[2] Waddington CH (1957) The strategy of the genes. A discussion of some aspects of theoretical biology. With an appendix by H. Kacser. London: George Allen and Unwin. 262 p.

[3] Zakharov VM (1989) Future prospects for population phenogenetics. Sov Sci Rev Section F, Phys Gen Biol Rev 4: 1–79.

[4] de Visser J, Hermisson J, Wagner GP, Meyers LA, Bagheri-Chaichian H, et al (2003) Perspective: evolution and detection of genetic robustness. Evolution 57: 1959–1972. 14575319 10.1111/j.0014-3820.2003.tb00377.x

[5] Masel J, Siegal ML (2009) Robustness: mechanisms and consequences. Trends Genet 25: 395–403. 19717203 10.1016/j.tig.2009.07.005 PMC2770586

[6] Jarosz DF, Taipale M, Lindquist S (2010) Protein homeostasis and the phenotypic manifestation of genetic diversity: principles and mechanisms. Annu Rev Genet 44: 189–216. 21047258 10.1146/annurev.genet.40.110405.090412

[7] Whitacre JM (2012) Biological robustness: paradigms, mechanisms, and systems principles. Front Genet 3: 67 doi:10.3389/fgene.2012.00067. 22593762 10.3389/fgene.2012.00067 PMC3350086

[8] Leamy LJ, Klingenberg CP (2005) The genetics and evolution of fluctuating asymmetry. Annu Rev Ecol Evol Syst 36: 1–21.

[9] Graham JH, Raz S, Hel-Or H, Nevo E (2010) Fluctuating asymmetry: methods, theory, and applications. Symmetry 2: 466–540.

[10] Costanzo M, Baryshnikova A, Bellay J, Kim Y, Spear ED, et al (2010) The genetic landscape of a cell. Science 327: 425–431. 20093466 10.1126/science.1180823 PMC5600254

[11] Lehner B (2010) Genes confer similar robustness to environmental, stochastic, and genetic perturbations in yeast. PLoS ONE 5: e9035. 20140261 10.1371/journal.pone.0009035 PMC2815791

[12] Dobzhansky T (1950) Genetics of natural populations. XIX. Origin of heterosis through natural selection in populations of *Drosophila pseudoobscura* . Genetics 35: 288–302. 15414931 10.1093/genetics/35.3.288 PMC1209487

[13] Lerner IM (1954) Genetic homeostasis. New York: Wiley. 134 p.

[14] Graham JH, Felley JD (1985) Genomic coadaptation and developmental stability within introgressed populations of *Enneacanthus gloriosus* and *E. obesus* (Pisces, Centrarchidae). Evolution 39: 104–114. 28563632 10.1111/j.1558-5646.1985.tb04083.x

[15] Milton CC, Huynh B, Batterham P, Rutherford SL, Hoffmann AA (2003) Quantitative trait symmetry independent of Hsp90 buffering: distinct modes of genetic canalization and developmental stability. Proc Natl Acad Sci U S A 100: 13396–13401. 14595030 10.1073/pnas.1835613100 PMC263825

[16] Milton CC, Batterham P, McKenzie JA, Hoffmann AA (2005) Effect of *E(sev)* and *Su(Raf) Hsp83* mutants and trans-heterozygotes on bristle trait means and variation in *Drosophila melanogaster* . Genetics 171: 119–130. 16183907 10.1534/genetics.104.038463 PMC1456505

[17] Debat V, Milton CC, Rutherford S, Klingenberg CP, Hoffmann AA (2006) Hsp90 and the quantitative variation of wing shape in *Drosophila melanogaster* . Evolution 60: 2529–2538. 17263114

[18] Sangster TA, Salathia N, Undurraga S, Milo R, Schellenberg K, et al (2008) HSP90 affects the expression of genetic variation and developmental stability in quantitative traits. Proc Natl Acad Sci U S A 105: 2963–2968. 18287065 10.1073/pnas.0712200105 PMC2268568

[19] Wagner A (2005) Distributed robustness versus redundancy as causes of mutational robustness. Bioessays 27: 176–188. 15666345 10.1002/bies.20170

[20] Edwards JS, Palsson BO (2000) Robustness analysis of the *Escherichia coli* metabolic network. Biotechnol Prog 16: 927–939. 11101318 10.1021/bp0000712

[21] Edwards JS, Palsson BO (2000) The *Escherichia coli* MG1655 in silico metabolic genotype: its definition, characteristics, and capabilities. Proc Natl Acad Sci U S A 97: 5528–5533. 10805808 10.1073/pnas.97.10.5528 PMC25862

[22] Félix MA, Wagner A (2008) Robustness and evolution: concepts, insights and challenges from a developmental model system. Heredity 100: 132–140. 17167519 10.1038/sj.hdy.6800915

[23] Siegal ML, Promislow DEL, Bergman A (2007) Functional and evolutionary inference in gene networks: does topology matter? Genetica 129: 83–103. 16897451 10.1007/s10709-006-0035-0

[24] Jeong H, Tombor B, Albert R, Oltvai ZN, Barabási AL (2000) The large-scale organization of metabolic networks. Nature 407: 651–654. 11034217 10.1038/35036627

[25] Khanin R, Wit E (2006) How scale-free are biological networks. J Comput Biol 13: 810–818. 16706727 10.1089/cmb.2006.13.810

[26] Przytycka TM, Yu YK (2004) Scale-free networks versus evolutionary drift. Comput Biol Chem 28: 257–264. 15548452 10.1016/j.compbiolchem.2004.07.001 PMC1680142

[27] Lima-Mendez G, Van Helden J (2009) The powerful law of the power law and other myths in network biology. Mol Bio Syst 5: 1482–1493. 10.1039/b908681a 20023717

[28] Keller EF (2005) Revisiting “scale-free” networks. Bioessays 27: 1060–1068. 16163729 10.1002/bies.20294

[29] Levy SF, Siegal ML (2008) Network hubs buffer environmental variation in *Saccharomyces cerevisiae* . PLoS Biol 6: e264. 18986213 10.1371/journal.pbio.0060264 PMC2577700

[30] Graham JH, Emlen JM, Freeman DC (2003) Nonlinear dynamics and developmental instability. In: Polak M, editor. Developmental instability: Causes and consequences. New York: Oxford University Press. pp. 35–50.

[31] Babbitt GA, Kiltie R, Bolker B (2006) Are fluctuating asymmetry studies adequately sampled? Implications of a new model for size distribution. Am Nat 167: 230–245. 16670983 10.1086/498621

[32] Fang Z, Wang J, Liu B, Gong W (2012) Double Pareto lognormal distributions in complex networks. In: Thai MT, Pardalos PM, editors. Handbook of optimization in complex networks: Theory and application. New York: Springer. pp. 55–80.

[33] Lu C, King RD (2009) An investigation into the population abundance distribution of mRNAs, proteins, and metabolites in biological systems. Bioinformatics 25: 2020–2027. 19535531 10.1093/bioinformatics/btp360

[34] Newman MEJ (2005) Power laws, Pareto distributions and Zipf's law. Contemp Phys 46: 323–351.

[35] Kuznetsov V, Knott G, Bonner R (2002) General statistics of stochastic process of gene expression in eukaryotic cells. Genetics 161: 1321–1332. 12136033 10.1093/genetics/161.3.1321 PMC1462190

[36] Reed WJ, Jorgensen M (2004) The double Pareto-lognormal distribution—a new parametric model for size distributions. Commun Stat-Theor M 33: 1733–1753.

[37] Barabási AL, Oltvai ZN (2004) Network biology: understanding the cell's functional organization. Nat Rev Genet 5: 101–113. 14735121 10.1038/nrg1272

[38] Formstecher E, Aresta S, Collura V, Hamburger A, Meil A, et al (2005) Protein interaction mapping: a *Drosophila* case study. Genome Res 15: 376–384. 15710747 10.1101/gr.2659105 PMC551564

[39] Yu H, Braun P, Yildirim MA, Lemmens I, Venkatesan K, et al (2008) High-quality binary protein interaction map of the yeast interactome network. Science 322: 104–110. 18719252 10.1126/science.1158684 PMC2746753

[40] Ohya Y, Sese J, Yukawa M, Sano F, Nakatani Y, et al (2005) High-dimensional and large-scale phenotyping of yeast mutants. Proc Natl Acad Sci U S A 102: 19015–19020. 16365294 10.1073/pnas.0509436102 PMC1316885

[41] Press WH, Teukolsky SA, Vetterling WT, Flannery BP (1992) Numerical recipes in C: The art of scientific computing. Cambridge, UK: Cambridge University Press. 994 p.

[42] Akaike H (1981) Likelihood of a model and information criteria. J Econometrics 16: 3–14.

[43] Kelly WP, Ingram PJ, Stumpf MPH (2011) The degree distribution of networks: statistical model selection. In: van Helden J, Toussaint A, Thieffry D, editors. Bacterial molecular networks. New York, New York: Springer. pp. 245–262. 10.1007/978-1-61779-361-5_13 22144157

[44] Posada D, Buckley TR (2004) Model selection and model averaging in phylogenetics: advantages of Akaike information criterion and Bayesian approaches over likelihood ratio tests. Syst Biol 53: 793–808. 15545256 10.1080/10635150490522304

[45] Barabási AL, Albert R (1999) Emergence of scaling in random networks. Science 286: 509–512. 10521342 10.1126/science.286.5439.509

[46] Jeong H, Mason SP, Barabasi AL, Oltvai ZN (2001) Lethality and centrality in protein networks. Nature 411: 41–42. 11333967 10.1038/35075138

[47] Barkai N, Leibler S (1997) Robustness in simple biochemical networks. Nature 387: 913–917. 9202124 10.1038/43199

[48] Bhalla US, Iyengar R (1999) Emergent properties of networks of biological signaling pathways. Science 283: 381–387. 9888852 10.1126/science.283.5400.381

[49] Albert R, Jeong H, Barabási AL (2000) Error and attack tolerance of complex networks. Nature 406: 378–382. 10935628 10.1038/35019019

[50] Newman MEJ, Barabási AL, Watts DJ (2006) The structure and dynamics of networks. Princeton, New Jersey: Princeton University Press. 624 p.

[51] Stumpf MPH, Wiuf C, May RM (2005) Subnets of scale-free networks are not scale-free: sampling properties of networks. Proc Natl Acad Sci U S A 102: 4221–4224. 15767579 10.1073/pnas.0501179102 PMC555505

[52] Ravasz E, Barabási AL (2003) Hierarchical organization in complex networks. Phys Rev E 67: 1–7. 10.1103/PhysRevE.67.026112 12636753

[53] Stumpf MPH, Ingram PJ (2005) Probability models for degree distributions of protein interaction networks. Europhys Lett 71: 152–158.

[54] Gerlee P, Lundh T, Zhang B, Anderson ARA (2009) Gene divergence and pathway duplication in the metabolic network of yeast and digital organisms. J R Soc Interface 6: 1233–1245. 19324678 10.1098/rsif.2008.0514 PMC2817152

[55] Albert R (2005) Scale-free networks in cell biology. J Cell Sci 118: 4947–4957. 16254242 10.1242/jcs.02714

[56] Guelzim N, Bottani S, Bourgine P, Képès F (2002) Topological and causal structure of the yeast transcriptional regulatory network. Nat Genet 31: 60–63. 11967534 10.1038/ng873

[57] Reed WJ (2002) On the rank-size distribution for human settlements. J Reg Sci 42: 1–17.

[58] Giesen K, Zimmermann A, Suedekum J (2010) The size distribution across all cities–double Pareto lognormal strikes. J Urban Econ 68: 129–137.

[59] Graham JH, Shimizu K, Emlen JM, Freeman DC, Merkel J (2003) Growth models and the expected distribution of fluctuating asymmetry. Biol J Linn Soc 80: 57–65.

[60] Turcotte DL, Rundle JB (2002) Self-organized complexity in the physical, biological, and social sciences. Proc Natl Acad Sci U S A 99: 2463–2465. 11875195 10.1073/pnas.012579399 PMC128561

[61] West GB, Woodruff WH, Brown JH (2002) Allometric scaling of metabolic rate from molecules and mitochondria to cells and mammals. Proc Natl Acad Sci U S A 99: 2473–2478. 11875197 10.1073/pnas.012579799 PMC128563

[62] Csermely P (2004) Strong links are important, but weak links stabilize them. Trends Biochem Sci 29: 331–334. 15236738 10.1016/j.tibs.2004.05.004

[63] van Dongen S, Møller AP (2007) On the distribution of developmental errors: comparing the normal, gamma, and log-normal distribution. Biol J Linn Soc 92: 197–210.

[64] Freeman DC, Graham JH, Emlen JM (1993) Developmental stability in plants: symmetries, stress and epigenesis. Genetica 89: 97–119.

[65] Freeman DC, Graham JH, Emlen JM, Tracy M, Hough RA, et al.. (2003) Plant developmental instability: new measures, applications, and regulation. In: Polak M, editor. Developmental instability: Causes and consequences. New York: Oxford University Press. pp. 367–386.

[66] Raz S, Graham JH, Hel-Or H, Pavlíček T, Nevo E (2011) Developmental instability of vascular plants in contrasting microclimates at ‘Evolution Canyon’. Biol J Linn Soc 102: 786–797.

[67] Raz S, Graham JH, Cohen A, de Bivort BL, Grishkan I, et al (2012) Growth and asymmetry of soil microfungal colonies from “Evolution Canyon,” Lower Nahal Oren, Mount Carmel, Israel. PLoS ONE 7: e34689. 22523554 10.1371/journal.pone.0034689 PMC3327715

[68] Palková Z (2004) Multicellular microorganisms: laboratory versus nature. EMBO Rep 5: 470–476. 15184977 10.1038/sj.embor.7400145 PMC1299056

[69] Mináriková L, Kuthan M, Ricicová M, Forstová J, Palková Z (2001) Differentiated gene expression in cells within yeast colonies. Exp Cell Res 271: 296–304. 11716542 10.1006/excr.2001.5379

