# Supplementary material for: Random Phenotypic Variation of Yeast (Saccharomyces cerevisiae) Single-Gene Knockouts Fits a Double Pareto-Lognormal Distribution
Source: PLoS One. 2012 Nov 6;7(11):e48964. doi: 10.1371/journal.pone.0048964 (PMC3490920; doi:10.1371/journal.pone.0048964)
Supplement: Appendix S1 — Errata in the original article on the double Pareto-lognormal distribution by Reed. (DOCX) [file pone.0048964.s001.docx]

**Appendix S1**

We found four errata in the original article by Reed [[36](#_ENREF_36)], and found it necessary to correct these before we could duplicate several of the figures in his paper.

**1.** Instead of $A(\theta,\nu,\tau)=\exp\left(\theta\nu+\alpha^{2}\tau^{2}/2\right)$, Eq. (9) in Reed should read $A(\theta,\nu,\tau)=\exp\left(\theta\nu+\theta^{2}\tau^{2}/2\right)$, a change from *α*^2^ to *θ*^2^ in the second term inside the exponential.

**2.** Instead of $G(y)=\Phi\left(\frac{y-\nu}{\tau}\right)-\varphi\left(\frac{y-\nu}{\tau}\right)\frac{\beta R\left(\alpha\tau-(y-\nu)/\tau\right)+\alpha R\left(\beta\tau+(y-\nu)/\tau\right)}{\alpha+\beta}$,

Eq. (15) in Reed [[36](#_ENREF_36)] should be $G(y)=\Phi\left(\frac{y-\nu}{\tau}\right)-\varphi\left(\frac{y-\nu}{\tau}\right)\frac{\beta R\left(\alpha\tau-(y-\nu)/\tau\right)-\alpha R\left(\beta\tau+(y-\nu)/\tau\right)}{\alpha+\beta}$,

a change of a plus sign to minus sign between the two terms in the numerator of the big fraction.

**3.** Instead of

$$F(x)=\Phi\left(\frac{\log x-\nu}{\tau}\right)-\frac{1}{\alpha+\beta}\left[\beta x^{-\alpha}A(\alpha,\nu,\tau)\Phi\left(\frac{\log x-v-\alpha\tau^{2}}{\tau}\right)+\alpha x^{\beta}A(-\beta,\nu,\tau)\Phi^{c}\left(\frac{\log x-v+\beta\tau^{2}}{\tau}\right)\right]$$

Eq. (23) in Reed [[36](#_ENREF_36)] should be

$$F(x)=\Phi\left(\frac{\log x-\nu}{\tau}\right)-\frac{1}{\alpha+\beta}\left[\beta x^{-\alpha}A(\alpha,\nu,\tau)\Phi\left(\frac{\log x-v-\alpha\tau^{2}}{\tau}\right)-\alpha x^{\beta}A(-\beta,\nu,\tau)\Phi^{c}\left(\frac{\log x-v+\beta\tau^{2}}{\tau}\right)\right]$$

This is just a single change of a plus sign to a minus sign between the two terms in the big brackets.

**4**. The text above Eq. (23) in Reed [[36](#_ENREF_36)] should be “. . . can be written as *F*(*x*) = *G*(log *x*)”, rather than “. . . can be written as *F*(*x*) = *G*(*e^x^*).”
